# Mechanism of action biomarkers predicting response to AKT inhibition in the I-SPY 2 breast cancer trial

**DOI:** 10.1038/s41523-020-00189-2

**Published:** 2020-10-02

**Authors:** Denise M. Wolf, Christina Yau, Julia Wulfkuhle, Lamorna Brown-Swigart, Rosa I. Gallagher, Mark Jesus M. Magbanua, Nick O’Grady, Gillian Hirst, I-SPY 2 TRIAL Investigators, Smita Asare, Debu Tripathy, Don Berry, Laura Esserman, A. Jo Chien, Emanuel F. Petricoin, Laura van ‘t Veer

**Affiliations:** 1 University of California, San Francisco, San Francisco, CA USA; 2 George Mason University, Fairfax, VA USA; 3 Quantum Leap Healthcare Collaborative, San Francisco, CA USA; 4 University of Texas, MD Anderson Cancer Center, Houston, TX USA; 5 Berry Consultants, LLC, Austin, TX USA

**Keywords:** Predictive markers, Breast cancer

## Abstract

The AKT inhibitor MK2206 (M) was evaluated in I-SPY 2 and graduated in the HER2+, HR−, and HR− HER2+ signatures. We hypothesized that AKT signaling axis proteins/genes may specifically predict response to M and tested 26 phospho-proteins and 10 genes involved in AKT-mTOR-HER signaling; in addition, we tested 9 genes from a previous study in the metastatic setting. One hundred and fifty patients had gene expression data from pretreatment biopsies available for analysis (M: 94, control: 56) and 138 had protein data (M: 87, control: 51). Logistic modeling was used to assess biomarker performance in pre-specified analysis. In general, phospho-protein biomarkers of activity in the AKT-mTOR-HER pathway appeared more predictive of response to M than gene expression or total protein biomarkers in the same pathway; however, the nature of the predictive biomarkers differed in the HER2+ and TN groups. In the HER2+ subset, patients achieving a pCR in M had higher levels of multiple AKT kinase substrate phospho-proteins (e.g., pmTOR, pTSC2). In contrast, in the TN subset responding patients had lower levels of AKT pathway phospho-proteins, such as pAKT, pmTOR, and pTSC2. Pathway mutations did not appear to account for these associations. Additional exploratory whole-transcriptome analysis revealed immune signaling as strongly associated with response to M in the HER2+ subset. While our sample size is small, these results suggest that the measurement of particular AKT kinase substrate phospho-proteins could be predictive of MK2206 efficacy in both HER2+ and TN tumors and that immune signaling may play a role in response in HER2+ patients.

## Introduction

The AKT/mammalian target of rapamycin (mTOR)/phosphoinositide-3 kinase (PI3K) signaling pathway plays a pivotal role in the development, survival, and proliferation of tumor cells, making it an attractive drug target. Activation of PI3K heterodimers requires the coupling of growth factor or ligand to growth factor receptor tyrosine kinases (RTKs) such as insulin-like growth factor I receptor (IGF1R) or HER family proteins. Activated PI3K phosphorylates phosphatidylinositol 4,5-bisphosphate (PIP2) to phosphatidylinositol 3,4,5-trisphosphate (PIP3); and PIP3 provides a docking site for AKT leading to its phosphorylation. AKT, a serine/threonine kinase, is the central mediator of the PI3K pathway. Phosphatase and tensin homolog (PTEN), a tumor suppressor, acts in opposition to activated PI3K by dephosphorylating PIP3–PIP2 and therefore lowering the level of activated (phospho-) AKT. Thus the level of signaling through this pathway is a function of the balance between PI3K kinase activity and PTEN phosphatase activity, which are in turn regulated by ligands, RTKs, and other molecules. Phosphorylated AKT controls cellular phenotype by activating the mTOR complex that regulates RNA translation, protein synthesis, cell growth, and autophagy, among other processes^1–3^.

A plethora of agents have been developed to inhibit this key cancer pathway by targeting AKT (e.g., MK2206, ipataserib, perifosine) or PI3K/mTOR (e.g., gefitinib, erlotinib, everolimus), along with dual PI3K/mTOR inhibitors designed to overcome compensatory resistance mechanisms. In addition to treating the (relatively rare) cancers with strong mutational drivers in this pathway, these drugs are viewed as promising partners in combination therapy with a variety of anticancer agents including taxanes, supported by preclinical evidence that they may help overcome resistance mechanisms. Recent clinical trials have shown a survival benefit to combination therapy in the metastatic setting^1^.

**Fig. 1:**
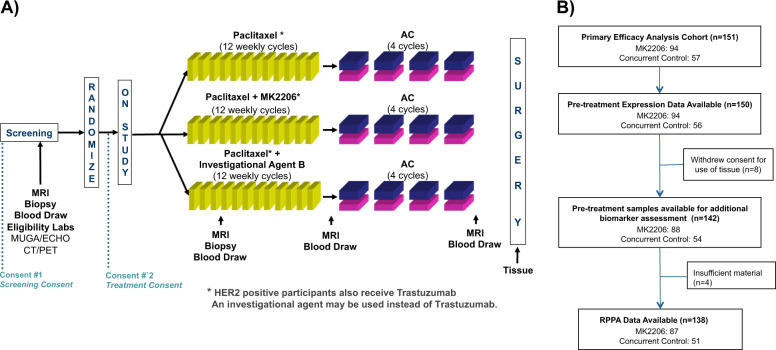
Trial design and data. **a** I-SPY 2 TRIAL schematic. **b** Consort diagram that outlines the number of patients included in the MK2206 and control arms of the I-SPY 2 TRIAL and those included in the subsequent analyses. RPPA reverse phase protein array.

MK2206, a clinically advanced AKT inhibitor, was recently evaluated in I-SPY 2, a multicenter phase 2 adaptive standing platform trial for women with *early stage*, locally advanced, aggressive breast cancer^4^. I-SPY 2 is designed to screen multiple experimental regimens in addition to standard neoadjuvant chemotherapy (NAC; Fig. 1a). This trial is adaptive, in that a patient randomized to receive experimental treatment is assigned preferentially to the arm where her cancer subtype is most likely to respond. Subtype is defined by hormone receptor (HR) status, HER2 status, and MammaPrint (MP) High 1/High 2 risk status (MP1/2), essentially a further stratification of the MP poor prognosis group (MP High) into high- and ultra-high-risk groups. The primary endpoint is pathologic complete response (pCR), i.e., no invasive cancer left in the breast or lymph nodes. The goal of I-SPY 2 is to identify (graduate) regimens with >85% predicted probability of succeeding in a 1:1 randomized 300-patient phase 3 trial where pCR is the endpoint and in the signatures defined by HR, HER2, and MP where the drug is most effective. The MK2206 arm was open for enrollment to all subtypes and was eligible for graduation in all ten signatures.

MK2206 successfully graduated in the HER2+, HR−, and HR− HER2+ signatures^4^, but not all patients within these subsets achieved a pCR in the MK2206 arm, and many outside these groups (i.e., triple negative (TN), HR+ HER2+, and HR+ HER2−) responded; for instance, the estimated pCR rate for TN patients was 40% in the MK2206 arm vs. 22% in control. There is a need for additional robust biomarkers that predict MK2206 sensitivity. Previous studies have proposed AKT/PI3K and HER pathway genes, proteins, and mutations/copy number alteration as possible markers of sensitivity to AKT inhibition, but their predictive performance in a variety of settings has been mixed. Supportive studies include PTEN small interfering RNA knockdown experiments in breast cancer cells showing increased AKT phosphorylation concordant with increased MK2206 sensitivity^5^ and in vitro lung and ovarian cancer studies demonstrating synergistic inhibition of AKT and epidermal growth factor receptor (EGFR)/HER2^6^. In the luminal breast cancer cell line MCF-7, phosphatidylinositol-4,5-bisphosphate 3-kinase catalytic subunit alpha (PIK3CA) but not AKT1 mutation increased sensitivity to MK2206^7^. However, analysis of the circulating DNA PIK3CA somatic mutation status in metastatic patients in a phase 1 trial did not support the hypothesis that tumors with PIK3CA mutations have improved responsiveness to MK2206^8^. A patient-derived xenograft study of basal breast cancer suggests that increased PI3K pathway activity, likely due to loss of PTEN expression, was a biomarker for mTOR inhibitor and AKT inhibitor combination therapy but not for MK2206 monotherapy^9^. Recent evidence has found that increased phospho-AKT correlates with worse prognosis in HER2+ breast cancer^10^ and predicts paclitaxel benefit^11^. Interestingly, activation of first-order AKT kinase substrates such as FOXO1 and FOXO3 has been shown to correlate with good prognosis in luminal cancers^12,13^.

Based on the hypotheses that, since MK2206 is an enzymatic inhibitor of AKT, response to MK2206 may be predicted by the relative pretreatment levels of phosphorylation of AKT kinase substrates, in pre-defined analyses we assessed 26 well-known proteins/phospho-proteins and 10 genes in the AKT-mTOR-HER pathway to test their association with pCR in the MK2206 arm in both HER2+ and HER2− subsets. We specifically used laser capture microdissection (LCM)-enriched tumor epithelium cell isolates from the clinical biopsy specimens as the sample input for all protein pathway activation analysis, which has been shown to be critical in accurate measurement of ubiquitously expressed and activated signaling proteins^14,15^. To the best of our knowledge, this work represents the first rigorous study of association between AKT-mTOR-HER pathway gene and direct measurement of AKT pathway activation using phospho-protein activation levels and response to MK2206 performed in early-stage breast cancer patients.

In previous exploratory whole-transcriptome analysis, we identified nine genes associated with response to MK2206 in vitro and in the metastatic setting in HER2+ patients^16^. While one of these genes (STARD3) is on the ERBB2 amplicon, the other eight are not considered members of the AKT signaling pathway. We also tested these genes in this study and performed exploratory association analyses using whole-transcriptome data and all available reverse phase protein array (RPPA) endpoints to identify additional predictive signals outside the AKT-mTOR-HER pathway.

## Results

### Expression levels of AKT-mTOR-HER family and phase 1b/cell line predictive genes do not specifically predict MK2206 sensitivity after adjusting for HR and HER2 status

**Fig. 2:**
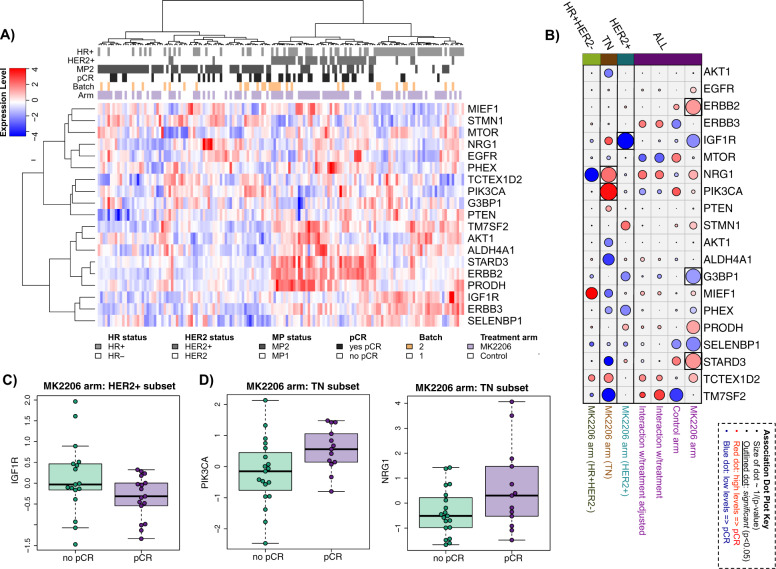
Analysis of pre-specified mechanism-of-action gene expression biomarkers for MK2206 response in patients enrolled in the I-SPY 2 TRIAL. **a** Clustered heatmap of genes (rows) and patient samples (columns), with samples annotated by HR/HER2 status (dark = positive), MP1/2 class (dark = MP2), response (dark = pCR), Agilent array batch (gold = second), and arm (purple = MK2206). **b** Association dotplot showing the level and direction of association between each gene (row) and pCR in the population/model as labeled (columns from right to left): MK2206 arm, control arm, interaction with treatment, interaction with treatment in a model adjusting for HR/HER2; and MK2206 arm limited to the HER2+, TN, and HR+ HER2− subsets. Key = red/blue dot indicates higher/lower levels ~ pCR; size of dot ~ strength of association (1/*p*), with dark outline indicating *p* < 0.05. **c**, **d** show boxplots for IGF1R in the HER2+ subset (**c**) and PIK3CA and NRG1 in the TN subset (**d**). See Supplementary Table 1 for association data.

The ten pre-defined AKT-mTOR-HER family signaling genes (AKT1, EGFR, ERBB2, ERBB3, NRG1, IGF1R, PIK3CA, PTEN, STMN1, and MTOR) and nine genes previously identified as associated with response in HER2+ metastatic patients (STARD3, TM7SF2, ALDH4A1, PRODH, SELENBP1, G3BP1, SMCR7L, TCTEXD2, and PHEX) co-cluster, as shown in the heatmap of Fig. 2a. In particular, genes on the ERBB2 amplicon ERBB2 and STARD3 are tightly correlated and associate with pCR in the MK2206 arm (likelihood ratio Looks good (LR) *p* < 0.05) but not in the control arm (rightmost two columns of pCR dotplot in Fig. 2b), consistent with MK2206 graduation in the HER2+ subtype. Also consistent with previous findings, G3BP1, a component of the RAS signaling pathway, associates with non-pCR in the MK2206 arm. However, biomarker × treatment interactions for these genes are not significant, and all three associations with response to MK2206 lose significance in a model adjusting for HR and HER2 status (Fig. 2b and Supplementary Table 1).

Within the HER2+ subset, the luminal marker IGF1R is associated with non-pCR in MK2206 (LR *p* < 0.05; Fig. 2b, third column from left, and boxplot in Fig. 2c); however, this association loses significance in a model adjusting for HR status. Within the TN subset, higher levels of NRG1 and PIK3CA, upstream activators of AKT, associate with pCR in the MK2206 arm (LR *p* < 0.05, Fig. 2b, second column from left, and boxplot in Fig. 2d). Though we were not able to evaluate whether these associations are specific to the MK2206 arm or also present in the control arm using data from I-SPY 2 due to the small sample size (Ctr: 3/23 pCR in TN), we assessed associations using data from another trial where patients received standard NAC (I-SPY 1 (GSE22226); *n* = 51 TN). Neither NRG1 nor PIK3CA associate with pCR in this cohort (LR *p* = 0.78 and 0.20, respectively), suggesting that these genes may be specific predictors of TN response to AKT inhibition. See Supplementary Table 1 for detailed pCR association results.

### AKT-mTOR-HER family protein signaling activation predicts MK2206 sensitivity in a subtype-specific manner

**Fig. 3:**
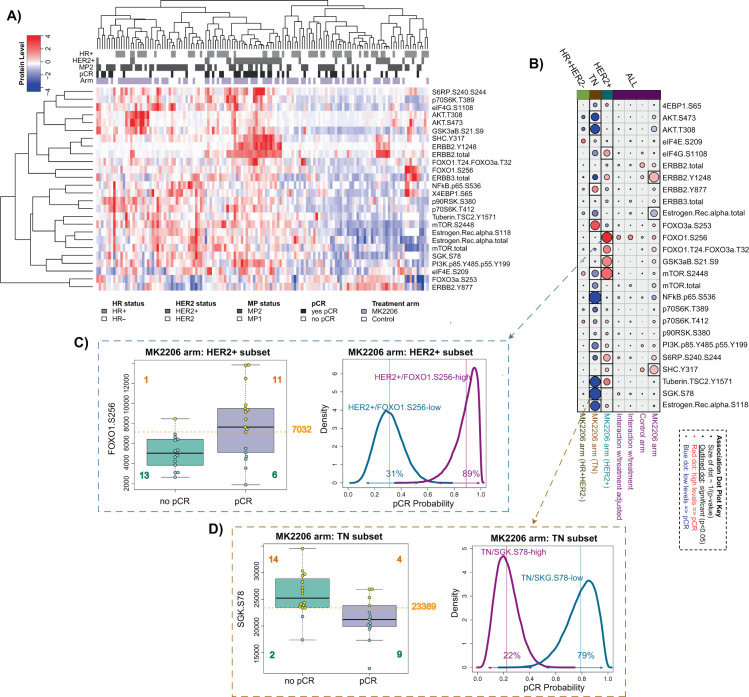
Analysis of pre-specified mechanism-of-action protein/phospho-protein RPPA biomarkers for MK2206 response in patients enrolled in the I-SPY 2 TRIAL. **a** Clustered heatmap of RPPA endpoints (rows) and patient samples (columns), with samples annotated by HR/HER2 status (dark = positive), MP1/2 class (dark = MP2), response (dark = pCR), and arm (purple = MK2206). **b** Association dotplot showing the level and direction of association between each protein endpoint (row) and pCR in the population/model as labeled (columns from right to left): MK2206 arm, control arm, interaction with treatment, interaction with treatment in a model adjusting for HR/HER2; and MK2206 arm within HER2+, TN, and HR+ HER2− subsets. Dot color reflects direction (red: higher in pCR; blue: lower in pCR) and dot size reflects strength (–log10(*p*)) of association with white background indicating *p* < 0.05. **c**, **d** show association data for example endpoints FOXO1 S256 in the HER2+ subset (**c**) and SGK S78 in the TN subset (**d**). Panels to the left show boxplots, with a golden broken line denoting optimal dichotomizing thresholds. Panels to the right show pCR probability distributions for subsets defined by the dichotomized biomarkers, where the estimated pCR rates are the means of each distribution as labeled (e.g., 89% estimated pCR for HER2+/FOXO1 S256-high vs. 31% for HER2+/FOXO1 S256-low). See Supplementary Table 2 for association data.

The heatmap visualization of the 26 pre-defined (qualifying) AKT-mTOR-HER family signaling proteins/phospho-proteins assayed from pretreatment, LCM-purified tumor epithelium (Fig. 3a) shows tight co-clustering of total ERBB2 protein and phospho-proteins ERBB2 Y1248 and SHC Y317, which are most highly expressed within the HER2+ subset. In the population as a whole, these two phospho-proteins are significantly associated with response in the MK2206 arm and not in the control arm (LR *p* < 0.05; pCR dotplot in Fig. 3b and Supplementary Table 2). As expected, these associations lose significance in a model adjusting for HER2 status, likely reflecting the high level of correlation between HER2 positivity, phospho-ERBB2, and phospho-SHC (Fig. 3a).

Estrogen receptor (ER) total protein clusters with phospho-ER and total mTOR (Fig. 3a), and higher levels are associated with non-response in MK2206, although this association loses significance in a model adjusting for receptor status (Fig. 3b and Supplementary Table 2).

In the HER2+ cohort, phosphorylation of seven AKT kinase substrates mTOR S2448, GSK3 S21/S9, FOXO1 S256, FOXO1 T24/FOXO3a T32, S6RP S240/S244, Tuberin/TSC2 Y1571, and eIF4G S1108 have significant (LR *p* < 0.05, actual *p* values in Supplementary Table 2) positive association with response in the MK2206 arm (Fig. 3b). FOXO1 S256 has the strongest association to response, as shown in the boxplot of Fig. 3c. Using the optimized dichotomizing cutpoint of 7032 identified through our cross-validation procedure, 39% of HER2+ patients are classified as FOXO1 S256-high. Bayesian analysis estimates a pCR rate of 89% for HER2+/FOXO1 S256-high patients compared to 31% for HER2+ patients with FOXO1 S256 below the dichotomizing threshold (Fig. 3c, right). Unfortunately, we were not able to evaluate whether these associations are specific to the MK2206 arm or also present in the control arm because of the small size of the latter (*n* = 9 HER2+ with RPPA in control).

In contrast, in TN, only two endpoints FOXO3a S253 (*p* = 0.031) and ERBB2 Y877 (*p* = 0.02) are positively associated with response (Fig. 3d and Supplementary Table 2). Surprisingly, most associations are in the negative direction, with low phospho-protein levels associated with pCR. AKT S473, AKT T308, ER alpha, mTOR, NFkB S536, and Tuberin/TSC2 Y1571 are negatively associated with MK2206 response. SGK S78, one of the most predictive biomarkers, is shown in the boxplots in Fig. 3d, along with the optimized dichotomizing cutpoint of 23,389 identified through cross-validation as described in “Methods.” Using this cutpoint, 38% of TN are SGK S78-low. TN/SGK S78-low patients have an estimated pCR rate of 79% compared to 22% for TN/SGK S78-high patients (Fig. 3d, right). As in the HER2+ subtype, the small number of TN pCRs in the control arm (3/22 with RPPA data) precluded assessment of biomarker × treatment interactions for this subset. All cutpoints derived from our cross-validation procedure, and Bayesian-estimated pCR probabilities in the resulting biomarker subsets, are considered exploratory and require validation in an external dataset. See Supplementary Table 2 for the complete set of pCR association results.

### Integrated biomarker heatmaps illustrate MK2206 response-based pathway modules

**Fig. 4:**
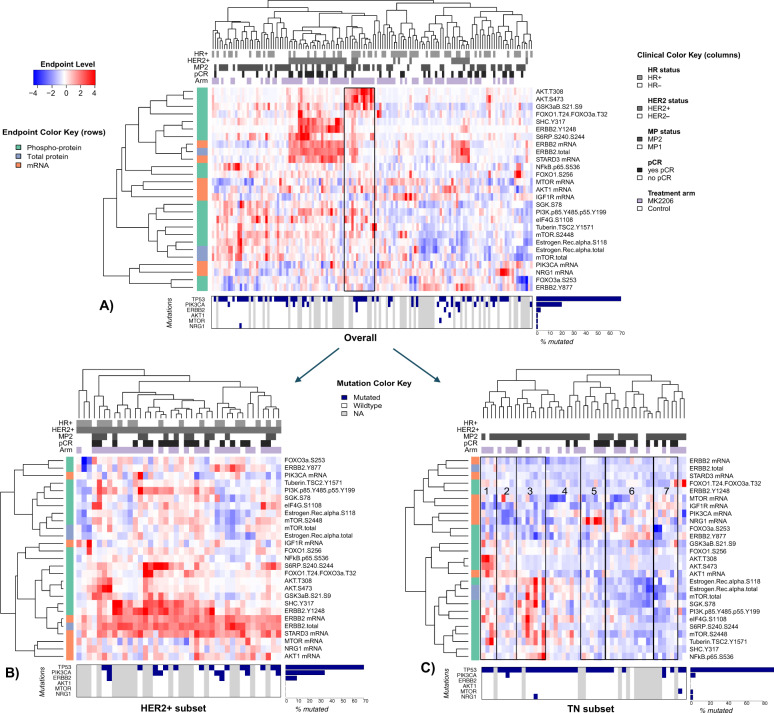
Integrated heatmaps showing the relationships between expression and protein/phospho-protein biomarkers. Heatmaps are shown for all patients (**a**) and for the HER2+ (**b**) and TN (**c**) subsets. Biomarkers represented include phospho-proteins associated with response to MK2206 and their associated mRNA and total protein levels (scaled to a median of 0 and standard deviation of 1). Mutation annotation strips at the bottom of each heatmap show the mutational status of TP53, PIK3CA, ERBB2, AKT1, MTOR, and NRG1 (blue = NS mutation; white = wild type; gray = missing); associated barplots to the right indicate %mutated in the population.

Across the population as a whole, unsupervised clustering of the above pCR-associated genes, total proteins, and phospho-proteins shows HER2 amplicon genes ERBB2 and STARD2 to be tightly co-expressed on the mRNA and total protein levels, with the highest levels in HER2+ patients. Phospho-proteins ERBB2 Y1248 and SHC Y317 form a cluster that can be either highly active or inactive within the HER2+ subset, with the former showing greater response (Fig. 4a, b). Phospho-AKT (T308 and S473) clusters tightly with phospho-GSK, mostly in non-responding HER2− patients, with activity levels that are distinct from AKT mRNA expression patterns (Fig. 4a, box).

In the TN subset (Fig. 4c), several signaling clusters are visible: HER/PI3K-related genes and proteins (top) and total and phospho-AKT (middle) and ER (luminal) related endpoints (bottom), including total/phospho-ER, total/phospho-mTOR, and phospho-PI3K. The signaling patterns appear relatively diverse. Most non-responding TNs are in the cluster on the left of the heatmap (Fig. 4c, boxes 1–4), characterized by higher luminal endpoints and/or AKT signaling and lower HER/PI3K levels. In contrast, the responding patients clustered on the right of the heatmap have lower signaling across the luminal nexus and higher signaling in one or more signals in the HER2/PI3K cluster that includes pERBB2, NRG1, and IGF1R/mTOR/PI3K mRNA but not the HER2-amplicon (Fig. 4c, boxes 5–7). Mutations in AKT1, PIK3CA/MTOR, and NRG1 are rare in TN patients and unlikely to be a major contributor to the extremely high levels of these endpoints in patients in boxes 1, 3, and 5 (Fig. 4c, mutation annotation strip).

### Exploratory whole-genome pathway enrichment analyses point to immune associations with response for HER2+ patients

Within the HER2+ subset, whole-transcriptome analysis identified 736 genes as associated with pCR in the MK2206 arm (LR *p* < 0.05). Pathway enrichment analysis using DAVID revealed immune pathways representing adaptive immune response, T/B cell immunity, chemokines, and dendritic cell signaling as the top most associated pathways (Supplementary Fig. 1a). Mean expression values of genes in these immune pathways are higher in responders than in non-responders, indicating that higher immune signaling associates with sensitivity (Supplementary Fig. 1b, c). As our HER2+ subset in the control arm is too small for comparative analysis (*n* = 10, 3 pCR), we assessed these pathways in other HER2+ patients receiving standard NAC (I-SPY 1 (GSE22226); *n* = 67, 30 pCR) and found that they did not predict pCR (LR *p* = 0.49 for Immune_GO_6955 and LR *p* = 0.35 for Immune_chemokine_GO_70098). This suggests that immune-infiltrated HER2+ tumors may be especially likely to respond to combination therapy that includes an AKT inhibitor.

Within the TN subset, despite association of 1031 genes with pCR in the MK2206 arm, DAVID pathway enrichment analysis of these data showed fewer enriched pathways with lower enrichment scores, suggesting a lower level of functional coherence among response-predictive genes compared to the HER2+ subset. The dominant pathway enrichment was a positive association between pCR and expression of histone genes/pathways (e.g., REACT_7970 Telomere Maintenance and chromatin organization (GO:0005325); Supplementary Fig. 1a, d). This histone pathway may specifically predict AKT-inhibition response, as it does not associate with pCR in TN patients receiving standard NAC (I-SPY 1 TRIAL (GSE22226); LR *p* = 0.66).

### Exploratory RPPA analysis of 118 protein/phospho-protein endpoints underscores divergent sensitivity signals in TN vs. HER2+ subsets

Association analysis of the entire set of 118 protein/phospho-proteins in the population as a whole yielded 12 protein/phospho-protein endpoints as associated with pCR in the MK2206 arm (LR *p* < 0.05; outlined dots in Supplementary Fig. 2, top row). Consistent with the qualifying biomarker analysis, the top five most predictive markers had higher levels in patients who achieved pCR and include multiple HER2 family endpoints.

In the TN subset, 27/118 protein endpoints associated with response in the MK2206 arm (LR *p* < 0.05; Supplementary Fig. 2, sixth row). Of these, all but two are negatively associated with response. In addition to previously described downstream AKT phosphorylation targets, these include D175-cleaved Caspase 3 and RTK-ROR1, both associated with apoptosis and possibly DNA repair deficiency.

Within HER2+ patients, there were 25/118 protein endpoints that associated with response in the MK2206 arm (LR *p* < 0.05; Supplementary Fig. 2, fifth row); with the exception of IGF1R, higher levels of these proteins/phospho-proteins were observed in responders. These include ten of the pre-specified endpoints, as well as the immune marker STAT5 Y694, consistent with our exploratory whole-transcriptome analysis. Additional kinases (e.g., ALK and pALK, pRET) and apoptosis markers (e.g., pBAD, caspases), cKIT, and pATR were identified as well.

## Discussion

Ideally, precision medicine should offer a menu of treatment–biomarker pairs to facilitate matching each breast cancer patient with the treatment most likely to save her life. To this end, early identification of predictive biomarkers has become increasingly important in a modern treatment landscape featuring an ever-growing number of anticancer agents targeting distinct cancer vulnerabilities. In the I-SPY 2 TRIAL, the MK2206 experimental arm was open to all HR/HER2 tumor subtypes, and gene expression arrays and a panel of proteomic/phospho-proteomic biomarkers were assayed in all pretreatment biopsy samples. These data present a unique opportunity to investigate the molecular correlates of response to AKT inhibition beyond receptor subtypes.

Due to sample size limitations, our biomarker work is more focused on hypothesis testing than discovery. This study primarily tests the hypothesis that mRNA, protein, or phospho-protein levels in the pathway targeted by the AKT inhibitor MK2206, the AKT/HER/mTOR network, specifically predict response to this agent. More mechanistically, the hypothesis is that breast cancers addicted to signaling through AKT, as evidenced by high levels of gene expression, protein, and especially phosphorylation (i.e., activation) of AKT and its direct kinase substrates along with downstream effector molecules, are sensitive to AKT kinase inhibition.

Analysis of 10 genes and 26 protein/phospho-proteins in the AKT/HER/mTOR pathway suggest that there is no single biomarker in this pathway that predicts response to MK2206 irrespective of receptor subtype, either at the gene expression level or the far more informative protein/phospho-protein level. Rather, predictive signals are highly subtype specific, with dramatic qualitative differences between the HER2+ and TN groups.

In the HER2+ subset, multiple AKT kinase substrate phospho-proteins associate with pCR in the MK2206 arm in the positive direction, meaning that, as hypothesized, higher levels associate with pCR and lower levels with non-response. These include pFOXO1, pGSK3aB, and pmTOR. The estimated pCR rate in the pFOXO1-high HER2+ population, using an optimal cutpoint, was 89%, compared with 31% with pCR in the pFOXO1-low HER2+ subset. Although these data are exploratory, this pCR rate was higher than the 48.3% of patients who had a CR observed in the graduating HER2+ subset.

In contrast, in the TN subset most AKT pathway phospho-proteins associating with response are negatively correlated, with lower rather than higher activity levels associated with pCR in the MK2206 arm. These include the phospho-proteins pAKT, pSGK, pmTOR, pTSC2, and pSGK. Using an optimized cutpoint, the estimated pCR rate in the pSGK-low TN population was 79%, compared with 22% with pCR in the pSGK-high TN subset and 40% in the TN subset as a whole, where MK2206 did not graduate.

Why is the direction of association in TNs the opposite of what we hypothesized, with AKT pathway phospho-proteins mostly being negatively correlated with pCR rather than positively associated as was the case in the HER2+ population? Though it may be tempting to fault this unexpected result as a technical artifact of the RPPA assay, we observed concordance in protein phosphorylation association with pCR with multiple independent members of the AKT-mTOR pathway in both the TN and HER2+ groups and also observed that AKT1 and mTOR expression is higher in the non-responders than in responders in the TN subset, though the association does not reach significance. The integrated heatmaps of the TNs in Fig. 4c may also provide some clues, as pmTOR, pPI3K, and to some extent pAKT co-cluster with ER, suggesting luminal-type biology known to be inherently chemo-resistant. Moreover, the luminal-block signals appear anti-correlated to signals in the HER/PI3K cluster that include pERBB2, NRG1, and PI3K mRNA. This may help elucidate the paradoxical finding that, on the mRNA level, high levels of PIK3CA and NRG1 associate with response, whereas on the phospho-protein level, high levels of pPI3K and mTOR/pmTOR associate with non-response (Figs. 2 and 3).

Another possibility is that the TN subset itself is a much more molecularly heterogeneous subset of tumors compared to the HER2+ cohort wherein the central role of AKT pathway activation may be less causal/actionable compared to HER2-driven AKT signaling. This molecular heterogeneity can be manifested by different inherent biochemical mechanisms, such as TN subsets characterized by differing levels of other RTK (e.g., ALK, EGFR, IGFR, MET, etc.) activation, activation of other HRs (such as androgen receptor and differing levels of ER signaling through membrane-initiated steroid signaling) and/or heterogeneous mTOR signaling feedback loops, which all may contribute to differential levels of AKT pathway activation. These various TN protein signaling subgroups, along with many other signaling events that could causally be important to tumor growth, can produce an overall mixed population containing some tumors with high levels of AKT activation that are not dependent on it for growth. Ultimately, further exploration of these AKT signaling biomarkers in TN will require much larger study sets of patients treated with AKT pathway inhibitors than was accrued in I-SPY 2 in order to better address the impact of inherent TN molecular heterogeneity.

Though sample sizes by receptor subtype are small, we also performed rudimentary whole-transcriptome and all-RPPA endpoint exploratory analysis. This analysis yielded the insight that high levels of immune signals associate with response in the HER2+ subset, perhaps due to synergy between MK2206 and trastuzumab, which is known to mobilize antitumoral immune recognition. Immune signals did not appear to associate with response in the TN group; rather, groups of chromatin-modifying and histone genes and pathways, and some DNA repair deficiency and apoptosis proteins, associated with MK2206 sensitivity. The phospho-protein-wide analysis also confirmed our observation in the more limited pre-specified biomarker set that HER2+ responding tumors are generally “warm,” with higher levels of predictive phospho-proteins, whereas TN responding tumors are mostly “cold,” with lower levels of predictive phospho-proteins (Fig. 3 and Supplementary Fig. 2).

The major caveats of these analyses concern sample size and balance. The adaptive design of the I-SPY 2 TRIAL efficiently and rapidly identifies agent–subtype combinations based on their estimated likelihood of phase III success but has the unfortunate consequence from a biomarker perspective of producing unbalanced groups with low patient numbers in each arm. Moreover, the subtype specificity of the biomarker associations further limits the analysis; for instance, the sample sizes of HER2+ and TN patient subsets in the control arm were too small or had too few pCRs to test for treatment interactions, so it will take additional data to evaluate whether the response predictive signals are specific to MK2206.

While our sample size is too small to draw definitive conclusions, our results suggest that the measurement of AKT kinase substrate phospho-proteins could be predictive of MK2206 clinical activity in both HER2+ and HER2− tumors, though the selection of proteins and their direction of association differ by subtype. These results will need to be validated in independent study sets in order to judge the significance of these initial findings.

## Methods

### Patients and trial schema

This correlative study involved 150 women with high-risk stage II and III early breast cancer who were enrolled in the multicenter, multi-arm, neo-adjuvant I-SPY 2 TRIAL (NCT01042379; IND 105139). Detailed descriptions of the design, eligibility, and study assessments in the I-SPY 2 trial have been reported previously^17,18^, including the efficacy of investigational agent MK2206^4^. MK2206 was active in the trial from September 15, 2012 to May 14, 2014. A total of 151 I-SPY 2 TRIAL patients were randomized to either the concurrent control (*n* = 57; paclitaxel followed by doxorubicin/cyclophosphamide; T → AC) or to the investigational arm MK2206 plus standard chemotherapy (n = 94; M + T → AC; see ref. ^4^ for patient characteristics). In both arms, HER2+ patients also received trastuzumab (Fig. 1a).

### Ethics

Institutional Review Boards at all participating institutions approved the protocol. All patients signed informed consent to allow research on and use of their biospecimen samples (see ref. ^4^ for details).

### Molecular assays/datasets

Pretreatment tumor samples were assayed using Agilent 44K (32,627; *n* = 119) or 32K (15,746; *n* = 31) expression arrays; and these data, as part of the first ~850 I-SPY 2 patient samples distributed over the two platforms, were combined into a single gene-level dataset after batch-adjusting the larger set using ComBat^19^. Previously published Agilent 44K gene expression microarray data from GEO Series GSE GSE22226 (I-SPY 1 TRIAL) was used in this manuscript as well, as supplemental “controls” receiving standard NAC. Whole-exome next-generation sequencing was performed by TGEN. Tumor mutations were identified from whole-exome sequencing data using three variant callers, Seurat v2.6, Strelka v1.0.13, and MuTect v1.1.4. Aberrations called by at least two or more callers were considered for subsequent analysis. In addition, LCM was performed to isolate tumor epithelium for signaling protein activation profiling by RPPA. RPPA samples were assayed on two arrays (all but six controls on one array), each containing hundreds of samples from different arms of the trial. To remove batch effects, we standardized each array prior to combining, by (1) sampling 5000 times, maintaining a receptor subtype balance equal to that of the first ~1000 patients (HR+ HER2−: 0.384, TN: 0.368, HR+ HER2+: 0.158, HR− HER2+: 0.09); (2) calculating the mean (mean) and mean (sd) for each RPPA endpoint; (3) *z*-scoring each endpoint using the calculated mean/sd from (2). The consort diagram with the number of evaluable patients for each molecular profiling analysis is shown in Fig. 1b. Details of the sample preparation and data processing are as previously described^20^.

### Statistical analysis

In our pre-specified analysis plan as previously summarized^20,21^, logistic regression is used to assess association with pCR in the control and MK2206-treated populations individually. Relative biomarker performance between arms (biomarker × treatment interaction) is assessed using a logistic model (pCR ~ treatment + biomarker + treatment × biomarker). Analysis is also performed adjusting for HR/HER2 (binary) status (pCR ~ treatment + biomarker + treatment:biomarker + HR+ HER2). Markers were analyzed individually; *p* values are descriptive.

In exploratory analysis, a cross-validation procedure was applied to selected endpoints associated with pCR in the MK2206 treatment arm of the trial to identify potential cutpoints for biomarker positivity. Twofold cross-validation was repeated 500 times with test and training sets balanced over pCR, using logistic models to assess association with response. The cutpoint with the minimum combined *p* value in the test sets (combined using the logit method^22^) was selected as “optimal,” after filtering to ensure it was also selected as optimal at least 10/500 times in the training sets. Dichotomized biomarkers are analyzed in a Bayesian framework using the MCMC simulation package rJAGS (Martyn Plummer (2019). rjags: Bayesian Graphical Models using MCMC. R package version 4–10. https://CRAN.R-project.org/package=rjags), based on I-SPY 2 data with the following model: pCR ~ HR+ HER2+ biomarker + treatment + treatment × HR+ treatment × HER2+ treatment × biomarker. Cutpoints and pCR probability estimates derived from this procedure are considered exploratory and require validation in an external dataset.

We also performed exploratory whole-transcriptome and phospho-proteome analysis, as above. Pathway enrichment analysis on gene expression was performed using the software tool DAVID employing Benjamini–Hochberg multiple testing correction^23^. All other analysis was performed in the R computing environment (R Core Team (2013). R: A language and environment for statistical computing).

### Reporting summary

Further information on research design is available in the Nature Research Reporting Summary linked to this article.

## Supplementary information

Supplementary Tables and Figures; plus consortium members

Reporting Summary Checklist FLAT

## Data Availability

The data generated and analyzed during this study are described in the following data record: 10.6084/m9.figshare.12490580^24^. De-identified molecular and clinical data used in this study have been deposited in NCBI’s Gene Expression Omnibus and are accessible through GEO SuperSeries accession number GSE150576^25^ and constituent Series accession numbers GSE149322 (gene expression data)^26^ and GSE150575 (RPPA protein/phospho-protein data)^27^. Linear transformation parameters (gene expression) and normalization parameters (mean, sd per RPPA endpoint) to transform raw to normalized data are available as supplemental files on Gene Expression Omnibus as well, along with the normalized data matrix used in our analysis (gene expression file = GSE149322_ExpDat_ISPY2_MK2206_n150.txt.gz).”

## Code availability

R scripts are available upon request.
