# Supplementary material for: Mechanism of action biomarkers predicting response to AKT inhibition in the I-SPY 2 breast cancer trial
Source: NPJ Breast Cancer. 2020 Oct 2;6:48. doi: 10.1038/s41523-020-00189-2 (PMC7532145; doi:10.1038/s41523-020-00189-2)
Supplement: Supplementary file 1 — Supplementary Tables and Figures; plus consortium members [file 41523_2020_189_MOESM1_ESM.pdf]

Mechanism of action biomarkers predicting response to AKT inhibition in the I-SPY2 breast cancer trial

Denise M Wolf[1]*, Christina Yau[1]*, Julia Wulfkuhle[2]*, Lamorna Brown-Swigart[1], Mark Magbanua[1], Nick O'Grady[1], Gillian Hirst[1], I-SPY 2 TRIAL Investigators, Smita Asare[3], Debu Tripathy[4], Don Berry[5], Laura Esserman[1], A. Jo Chien[1], Emanuel F. Petricoin III[2], Laura van 't Veer[1]

	Population as a whole with gene expression (n=150: 94 in MK2206, 56 in Ctr)								HER2+ subset (n=34 in MK2206)*		TN subset (n=32 in MK2206)*		HR+HER2- subset (n=28 in MK2206)*	
	Genes	MK2206 arm: OR/unit inc	MK2206 arm: LR p	MK2206 arm: LR p (adjusting for HR and HER2)	Cont arm: OR/unit inc	Cont arm: LR p	Biomarker x treatment int (LR p)	Biomarker x treatment int (adjusted for HR and HER2)	MK2206 arm: OR/unit inc	MK2206 arm: LR p	MK2206 arm: OR/unit inc	MK2206 arm: LR p	MK2206 arm: OR/unit inc	MK2206 arm: LR p
AKT/MTOR signaling axis	AKT1	0.949	0.803	0.855	0.944	0.887	0.967	0.899	0.986	0.97	0.586	0.175	0.912	0.849
	EGFR	1.23	0.305	1.02	1.01	0.985	0.66	0.664	1.02	0.949	1.21	0.698	0.884	0.806
	EGFR	1.23	0.305	1.09	1.01	0.985	0.66	0.664	1.02	0.949	1.21	0.698	0.884	0.806
	**ERBB2**	1.51	**0.0464**	1.15	1.47	0.329	0.964	0.927	1.32	0.644	1.17	0.836	0.88	0.916
	ERBB3	1.01	0.977	0.854	0.596	0.155	0.226	0.237	1.02	0.963	1.19	0.74	1.27	0.648
	**IGF1R**	0.628	0.0678	0.924	0.779	0.522	0.656	0.88	0.259	**0.0356**	2.03	0.2	0.722	0.52
	MTOR	0.905	0.652	1.33	1.69	0.157	0.144	0.213	1.09	0.807	0.747	0.462	0.781	0.659
	**NRG1**	1.37	0.153	1.2	0.742	0.444	0.182	0.17	0.765	0.626	1.79	**0.0457**	0.166	0.0592
	**PIK3CA**	1.15	0.481	1.14	1.99	0.18	0.428	0.267	0.857	0.568	2.62	**0.0303**	1.18	0.782
	PTEN	1.06	0.774	1.32	1.03	0.952	0.914	0.84	0.917	0.821	1.43	0.318	1.08	0.89
	STMN1	1.28	0.226	0.855	1.28	0.552	0.982	0.816	1.78	0.155	1.04	0.922	0.888	0.823
Phase 1b study genes	ALDH4A1	1.03	0.898	0.962	0.802	0.578	0.619	0.441	1.23	0.588	0.443	0.0937	1.3	0.643
	**G3BP1**	0.663	**0.0481**	0.78	0.979	0.963	0.429	0.725	0.611	0.151	0.999	0.998	0.692	0.479
	MIEF1	1.2	0.381	1.08	0.887	0.753	0.491	0.436	1.14	0.661	0.54	0.185	2.84	0.0892
	PHEX	0.809	0.274	0.7	0.795	0.681	0.974	0.71	0.608	0.122	0.654	0.232	0.894	0.827
	PRODH	1.44	0.067	1.3	1.04	0.921	0.499	0.584	1.39	0.287	0.97	0.949	1.43	0.662
	SELENBP1	0.614	0.0558	0.768	0.629	0.147	0.908	0.534	0.668	0.411	0.771	0.502	0.52	0.409
	**STARD3**	1.52	**0.0427**	1.15	1.71	0.174	0.785	0.553	1.37	0.504	0.131	0.15	0.651	0.773
	TCTEX1D2	1.34	0.161	1.47	0.831	0.65	0.304	0.255	1.05	0.907	1.71	0.151	1.68	0.265
	TM7SF2	0.971	0.888	0.723	0.444	0.0659	0.117	0.313	0.983	0.965	0.363	0.0642	0.564	0.487

Supplementary Table 1. pCR association analysis results for pre-specified mechanism-of-action genes, in the population as a whole and in receptor subsets. P-values < 0.05 are in bold face; red denotes markers where higher levels associate with pCR; and blue denotes markers where higher levels associate with non-response. *The small size and/or low number of pCR in the control group within receptor subsets precluded analysis (Ctr for HER2+: 3/10 pCR; TN: 3/24 pCR; HR+HER2+: 3/22 pCR).

Population as a whole with RPPA (n=138: 87 in MK2206, 51 in Ctr)								HER2+ subset (n=31 in MK2206)*		TN subset (n=29 in MK2206)*		HR+HER2- subset (n=27 in MK2206)*	
AKT/MTOR signaling axis phospho-proteins	MK2206 arm: OR/unit inc	MK2206 arm: LR p	MK2206 arm: LR p (adjusting for HR and HER2)	Cont arm: OR/unit inc	Cont arm: LR p	Biomarker x treatment int (LR p)	Biomarker x treatment int (adjusted for HR and HER2)	MK2206 arm: OR/unit inc	MK2206 arm: LR p	MK2206 arm: OR/unit inc	MK2206 arm: LR p	MK2206 arm: OR/unit inc	MK2206 arm: LR p
4EBP1.S65	0.824	0.581	0.491	0.96	0.887	0.737	0.758	2.55	0.25	0.441	0.148	0.891	0.885
**AKT.S473**	0.747	0.175	0.196	0.963	0.952	0.719	0.737	1.04	0.904	0.147	**0.021**	0.293	0.19
**AKT.T308**	0.658	0.0684	0.064	1.3	0.814	0.561	0.524	1.23	0.696	0.125	**0.011**	0.288	0.193
eIF4E.S209	1.2	0.407	0.944	0.863	0.716	0.469	0.843	1.29	0.45	0.669	0.3	3.2	0.14
**eIF4G.S1108**	1.18	0.465	0.983	1.34	0.386	0.748	0.517	2.22	**0.047**	0.406	0.0666	1.04	0.96
ERBB2.total	1.33	0.166	0.35	1.84	0.0996	0.442	0.422	0.606	0.333	0.37	0.733	2.18	0.695
**ERBB2.Y1248**	1.77	**0.00904**	0.186	1.54	0.222	0.741	0.652	1.74	0.0783	3.23E-05	0.0773	0.0107	0.512
**ERBB2.Y877**	1.29	0.292	0.446	1.05	0.892	0.622	0.549	0.626	0.22	2.66	**0.0496**	1.68	0.369
ERBB3.total	1.4	0.25	0.524	0.949	0.863	0.349	0.671	1.7	0.287	0.596	0.487	1.37	0.589
**Estrogen.Rec.alpha.total**	0.59	**0.0365**	0.185	0.865	0.668	0.379	0.706	1.15	0.765	0.34	0.0579	0.599	0.265
**FOXO3a.S253**	1.38	0.261	0.274	1.16	0.623	0.688	0.491	0.524	0.243	5.21	**0.0126**	1.24	0.693
**FOXO1.S256**	1.93	0.163	0.321	0.74	0.379	0.0991	0.14	11.2	**0.0072**	0.687	0.709	0.616	0.655
**FOXO1.T24.FOXO3a.T32**	1.35	0.125	0.569	1.47	0.518	0.894	0.933	2.9	**0.0258**	0.749	0.361	2.26	0.501
**GSK3aB.S21.S9**	1.31	0.18	0.17	1.23	0.653	0.908	0.872	2.84	**0.00937**	0.77	0.524	1.09	0.825
**mTOR.S2448**	1.23	0.346	0.288	1.18	0.634	0.92	0.62	3.84	**0.00425**	0.39	**0.0379**	1.82	0.166
mTOR.total	0.683	0.148	0.296	1	0.998	0.357	0.506	1.17	0.76	0.396	0.0612	0.805	0.708
**NFkB.p65.S536**	0.552	0.118	0.0604	0.773	0.439	0.537	0.386	1.55	0.533	0.126	**0.00327**	0.571	0.557
p70S6K.T389	1.16	0.467	0.675	1.06	0.904	0.852	0.743	1.25	0.393	0.249	0.0867	2.33	0.386
p70S6K.T412	1.29	0.294	0.644	1.01	0.97	0.528	0.644	1.55	0.23	0.476	0.155	2.25	0.231
p90RSK.S380	1.09	0.742	0.907	0.881	0.724	0.63	0.484	1.76	0.281	0.579	0.259	1.23	0.702
**PI3K.p85.Y485.p55.Y199**	1.24	0.302	0.792	1.48	0.323	0.701	0.704	1.52	0.185	0.319	**0.0394**	1.26	0.7
**S6RP.S240.S244**	1.42	0.106	0.432	1.01	0.973	0.43	0.357	2.05	**0.036**	0.267	0.0956	1.18	0.835
**SHC.Y317**	1.67	**0.0172**	0.252	1.95	0.152	0.76	0.851	1.41	0.18	0.871	0.898	2.11	0.758
**Tuberin.TSC2.Y1571**	0.859	0.529	0.403	1	0.995	0.779	0.868	3.19	**0.0434**	0.14	**0.00607**	0.77	0.76
**SGK.S78**	0.836	0.508	0.262	1.15	0.63	0.423	0.308	1.01	0.979	0.126	**0.00352**	1.49	0.534
**Estrogen.Rec.alpha.S118**	0.795	0.432	0.412	0.963	0.901	0.653	0.723	1.78	0.22	0.184	**0.00988**	0.803	0.735

Supplementary Table 2. pCR association analysis results for pre-specified RPPA endpoints, in the population as a whole and in receptor subsets. P-values < 0.05 are in bold face; red denotes markers where higher levels associate with pCR; and blue denotes markers where higher levels associate with non-response. *The small size and/or low number of pCR in the control group within receptor subsets precluded analysis (Ctr for HER2+: 3/9 pCR; TN: 3/22 pCR; HR+HER2+: 3/20 pCR).

A)

Category	Term	Count	PValue	Genes	Fold Enrichment	Benjamini	FDR
**MK2206 response pathway enrichments - HER2+ subset**							
GOTERM_BP_DIRECT	GO:0002250~adaptive immune response	20	7.77E-07	PIK3CG, GPR183, SIT1, CD1C, KLRK1, CD1B, TNFRSF17, CTSS, SLAMF7, CLEC10A, CD1E, TRAT1, PRKCB, BTK, BTLA, SH2D1A, CD79B, CD79A, CTSH, PAG1	3.9259	0.0019	0.0014
GOTERM_BP_DIRECT	GO:0006955~immune response	35	3.74E-06	C7, GPR183, ENPP2, IFITM2, IFITM3, IL18, CD70, HLA-DMB, IGF1R, CCL23, CXCR5, CCL21, HLA-DOA, THBS1, APLN, LTB, CD27, CR2, LTBR, CMKLR1, IL1RN, CCL19, CTSS, CCL18, CD1E, IGSF6, CCR8, TNFSF10, CCR6, CCL14, CCR2, IRF8, CD79B, TNFAIP1, ADAMDEC1	2.4152	0.0046	0.0066
GOTERM_BP_DIRECT	GO: 0070098~chemokine-mediated signaling pathway	13	4.86E-06	CCR8, CCL23, CCL14, CCR6, CXCR5, CMKLR1, CXCR4, CCL21, CCR2, CCL19, XCL1, CCL18, CCL17	5.3194	0.0040	0.0086
GOTERM_BP_DIRECT	GO:0007165~signal transduction	70	5.42E-06	GNA14, PPARG, FST, PDE3B, GJA1, FGF13, CD53, RASSF7, ARHGAP15, CD48, ANK1, PPP1R1A, PITPNC1, GKAP1, DLG5, PRKACB, APLN, NRG2, LTB, PAG1, LTBR, SIT1, TNFRSF17, CLIC2, TRAT1, PLAUR, PRKCB, JUP, CD38, CCR6, PLEKHH3, RIN1, PDE9A, AKAP8, INPP4A, EXT2, TRAF1, INPP1, OSTF1, PLCXD3, SPOCK2, KLRK1, CD70, SPOCK1, MDK, STAT6, IGF1R, TAGAP, CCL23, BCL11B, PDE1A, RASGRP2, HLA-DOA, TRAF4, PIK3R1, HPGDS, MRC1, PHB, DPYSL5, DRG2, SLAMF1, CCL18, TRIM55, P2RX4, LYVE1, TNFSF10, PNOC, CD79B, XCL1, GFRA3	1.7516	0.0033	0.0095
GOTERM_BP_DIRECT	GO:0002407~dendritic cell chemotaxis	7	1.44E-05	PIK3CG, GPR183, CCR6, CXCR4, CCL21, CCR2, CCL19	11.9625	0.0070	0.0253
GOTERM_BP_DIRECT	GO:0019722~calcium-mediated signaling	10	5.26E-05	CCR6, CXCR4, NCALD, PLCG2, HTR2B, LRRK2, RCAN3, SELE, BTK, MCTP1	5.6965	0.0212	0.0925
**MK2206 response pathway enrichments - TN subset**							
REACTOME_PATHWAY	REACT_7970:Telomere Maintenance	14	5.14E-06	HIST1H2AC, HIST1H2BB, HIST1H2BC, HIST1H2BD, HIST1H2BE, HIST1H2BG, POLE, HIST1H2BH, HIST1H2BO, RFC3, HIST1H2BM, HIST1H2BK, HIST1H2BL, HIST1H2BI, PRIM2, HIST1H2AK, HIST1H2AJ	4.6168	0.0003	0.0051
GOTERM_BP_FAT	GO:0006325~chromatin organization	41	3.15E-05	ING5, MORF4L1, SUPT3H, HIST1H2AC, HP1BP3, SETD1A, VPS72, TSPYL1, HIST1H2BO, CHD8, HIST1H2BM, CHD7, HIST1H2BK, PRMT7, HIST1H2BL, HIST1H2BI, GPX4, PBRM1, ACTL6A, KDM5C, CHD3, SETDB1, HIST1H2BB, HIST1H2BC, HIST1H2BD, HIST1H2BE, C11ORF30, HIST1H2BG, HIST1H2BH, ARID1A, BANP, ARID1B, TAF6L, SUV39H2, DOT1L, RNF8, TAF10, SAP130, SMARCC2, HIST1H2AK, RUVBL1, HIST1H2AJ, RNF20, RNF40	2.0156	0.0844	0.0563

B)

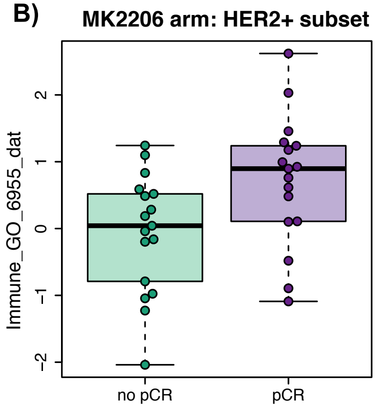

OR=2.64; LR p=0.0147

C)

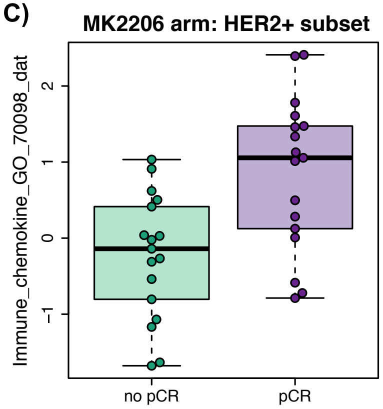

OR=3.68; LR p=0.0012

D)

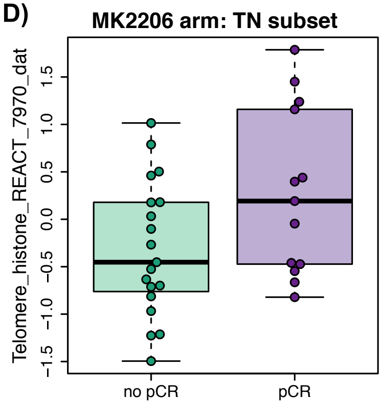

OR=2.65; LR p=0.039

Supplementary Figure 1. Whole genome exploratory pathway enrichment analysis of responders within the HER2+ and TN patient subsets, respectively, in the MK2206 arm. A) Pathway enrichment table of genes associated with pCR in the MK2206 arm using DAVID, top hits, for HER2+ patients (top) and TN patients (bottom). Box plots in (B) and (C) show the mean expression level of genes in the top two enriched (immune) pathways in pCR vs. non-pCR within the HER2+ subset. D) Box plot showing the mean expression level of genes in the top enriched (telomere maintenance/histone) pathway in pCR vs. non-pCR patients within the TN subset.

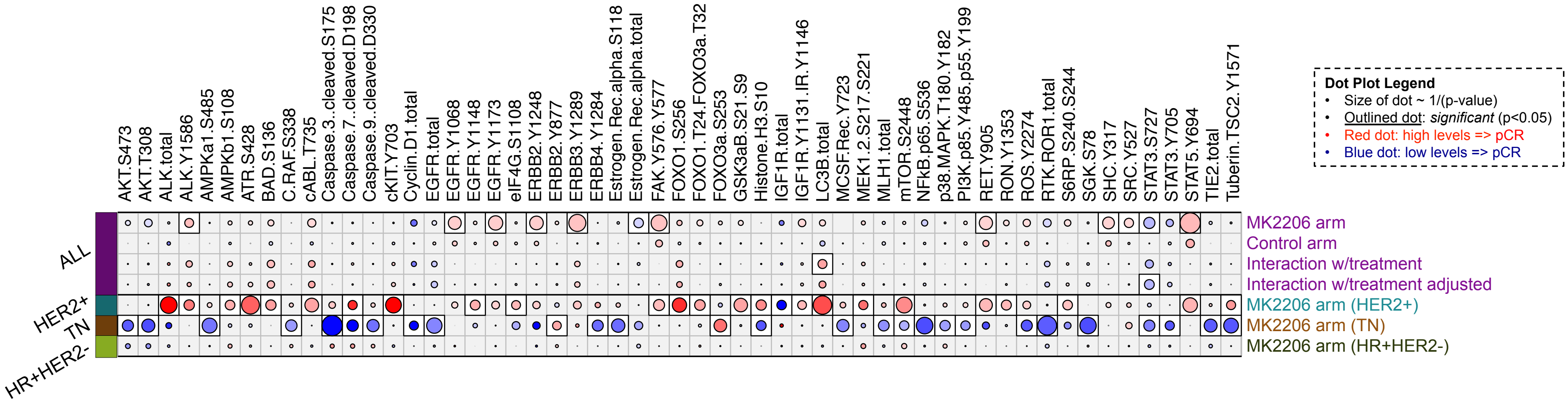

Supplementary Figure 2. Exploratory analysis of 118-endpoint RPPA dataset (115/118 with sufficient data for analysis). This association dot-plot shows the level and direction of association between each protein endpoint (column) and pCR in the MK2206 arm in patient groups as labeled (rows from top to bottom): all patients, HER2+, TN, and HR+HER2-. Key=red/blue dot indicates higher/lower levels ~ pCR; size of dot ~ strength of association (1/p), with dark outline indicating p<0.05. Only endpoints (columns) with at least one association with (uncorrected) p<0.05 are shown.

Supplementary Acknowledgement

We would like to acknowledge the following investigators, staff and sites for their contributions to the I-SPY 2 TRIAL during the period patients were being randomized to MK2206:

Note: Names in bold are site principal investigators, and names that appear in brackets no longer reside at that specific institution.

Liu, M.[6], Dillis, C.[6], Donnelly, R.[6], Harrington, T.[6], Isaacs, C.[6], Kallakury, B.[6], Lynce, F.[6], Oppong, B.[6], Pohlmann, P.[6], Tousimis, E.[6], Warren, R.[6], Willey, S.[6], Wong, J.[6], Zeck, J.6, **Albain, K.[7],** Bartolotta, M.[7], Bova, D.[7], Brooks, C.[7], Busby, B.7, Czaplicki, K.[7], Duan, X.[7], Gamez, R.[7], Ganesh, K.[7], Gaynor, E.[7], Godellas, C.[7], Grace-Louthen, C.[7], Kuritza, T.[7], Lo, S.[7], Nagamine, A.[7], Perez, C.[7], Robinson, P.[7], Rosi, D.[7], Vaince, F.[7], Ward, K.[7], Choquette, K.[8], **Edmiston, K.[8],** Gallimore, H.[8], McGovern, J.[8], Mokarem, K.[8], Pajaniappan, M.[8], Rassulova, S.[8], Scott, K.[8], Sherwood, K.[8], Wright, J.[8], Anderson, K.[9], Gray, R.[9], Myers, S.[9], **Northfelt, D.[9],** Pockaj, B.[9], Roedig, J.[9], Wasif, N.[9], Arens, A.[10], **Boughey, J.[10],** Brandt, K.[10], Carroll, J.[10], Chen, B.[10], Connors, A.[10], Degnim, A.[10], Farley, D.[10], Greenlee, S.[10], Haddad, T.[10], Hieken, T.[10], Hobday, T.[10], Jakub, J.[10], Liberte, L.[10], Liu, M.[10], Loprinzi, C.[10], Menard, L.[10], Moe, M.[10], Moynihan, T.[10], O'Sullivan, C.[10], Olson, E.[10], Peethambaram, P.[10], Ruddy, K.[10], Russell, B.[10], Rynearson, A.[10,] Smith, D.[10], Visscher, D.[10], Windish, A.[10], Bengtson, H.[11], Bucher, J.[11], **Chui, S.[11],** Gilbert-Ghormley, B.[11], Hampton, R.[11], Kemmer, K.[11], Kurdyla, D.[11], Nauman, D.[11], Spear, J.[11], Wilson, A.[11], Beatty, D.[12], Dawson, P.[12], Ellis, E.[12], Fer, M.[12], Hanson, J.[12], Goetz, M.[12], Haddad, T.[12], Iriarte, D.[12], **Kaplan, H.[12],** Porter, B.[12], Rinn, K.[12], Thomas, H.[12], Thornton, S.[12], Tickman, R.[12], Varghis, N.[12], Caterinichia, V.[13], Delos Santos, J.[13], Falkson, C.[13], **Forero, A.[13],** Krontiras, H.[13], Vaklavas, C.[13], Wei, S.[13], Bauland, A.[14], Inclan, L.[14], Lewallen, D.[14], Powell, A.[14], Roney, C.[14], Schmidt, K.[14], **Viscusi, R.[14],** Wright, H.[14], Blair, S.[15], Boles, S.[15], Bykowski, J.[15], Datnow, B.[15], Densley, L.[15], Eghtedari, M.[15], Genna, V.[15], Hasteh, F.[15], Helsten, T.[15], Kormanik, P.[15], Ojeda- Fournier, H.[15], Onyeacholem, I.[15], Parker, B.[15], Podsada, K.[15], Schwab, R.[15], **Wallace, A.[15],** Yashar, C.[15], Alvarado, M.[1], Au, A.[1], Balassanian, R.[1], Benz, C.[1], (Buxton, M.)[1], Chen, Y.[1], **Chien, J.[1],** D'Andrea, C.[1], (Davis, S.)[1], Esserman, L.[1], Ewing, C.[1], Goga, A.[1], Hirst, G.[1], Hwang, M.[1], Hylton, N.[1], Joe, B.[1], (Lyandres, J.)[1], Kadafour, M.[1], Krings, G.[1], Melisko, M.[1], Moasser, M.[1], Ngo, Z.[1], Park, J.[1], Price, E.[1], Rugo, H.[1], van't Veer, L.[1], Wolf, D.[1,] Wong, J.[1], Yau, C.[1], Abe, H.[16], Jaskowiak, N.[16], **Nanda, R.[16],** Olopade, F.[16], Schacht, D.[16], Borges, V.[17], Colvin, T.[17], Diamond, J.[17], **Elias, A.[17],** Finlayson, C.[17], Fisher, C.[17], Hardesty, L.[17], Kabos, P.[17], Kounalakis, N.[17], Mayordomo, J.[17], McSpadden, T.[17], Murphy, C.[17], Rabinovitch, R.[17], Sams, S.[17], Shagisultanova, E.[17], Beckwith, H.[18], Blaes, A.[18], Emory, T.[18], (Haddad, T.)[18], Hui, J.[18], Klein, M.[18], Kuehn-Hajder, J.[18], Nelson, M.[18], Potter, D.[18], Tuttle, T.[18], **Yee, D.[18],** Zera, R.[18], Bayne, L.[19], Bradbury, A.[19], Clark, A.[19], **DeMichele, A.[19],** Domchek, S.[19], Fisher, C.[19], Fox, K.[19], Frazee, D.[19], Lackaye, M.[19], Matro, J.[19], McDonald, E.[19], Rosen, M.[19], Shah, P.[19], Tchou, J.[19], Volpe, M.[19], Alvarez, R.[4], Barcenas, C.[4], Booser, D.[4], Brewster, A.[4], Brown, P.[4], **Gonzalez-Angulo, A.[4],** Ibrahim, N.[4], Karuturi, M.[4], Koenig, K.[4], **Moulder, S.[4],** Murray, J.[4], Murthy, R.[4], Pusztai, L.[4], Saigal, B.[4], Symmans, W.[4], Tripathy, D.[4], Theriault, R.[4], Ueno, N.[4], Valero, V.[4], Brown, M.[20], Carranza, M.[20], Flores, Y.[20], Lang, J.[20], Luna, A.[20], Perez, N.[20], Watkins, K.[20], Armstrong, S.[21], Boyd, C.[21], Chen, L.[21], Clark, V.[21], Frankel, A.[21], **Euhus, D.[21],** Froehlich, T.[21], Goudreau, S.[21], Haley, B.[21], Harker-Murray, A.[21], Klemow, D.[21], Leitch, A.[21], Leon, R.[21], Li, H.[21], Morgan, T.[21], Qureshi, N.[21], Rao, R.[21], Reeves, M.[21], Rivers, A.[21], Sadeghi, N.[21], Seiler, S.[21], Staves, B.[21], Tagoe, V.[21], Thomas, G.[21], Tripathy, D.[21], Unni, N.[21], Weyandt, S.[21], Wooldridge, R.[21], Zuckerman, J.[21], _**Korde, L.[22],** Griffin, M.[22], Butler, B.[22], Cundy, A.[22], Rubinstein, L.[22], Hixson, C.[22]

[6]Georgetown University, Washington, DC; [7]Loyola University Chicago Medical Center, Chicago, IL; [8]Inova Fairfax Hospital, Falls Church, VA; [9]Mayo Clinic, AZ; [10]Mayo Clinic, Rochester, MN; [11]Oregon Health and Science University, Portland, OR; [12]Swedish Cancer Institute, Seattle, WA; [13]University of Alabama, Birmingham, AL; [14]University of Arizona, Tucson, AZ; [15]University of California, San Diego, La Jolla, CA; [16]University of Chicago, Chicago, IL; [17]University of Colorado, Denver, CO; [18]University of Minnesota, Minneapolis, MN; [19]University of Pennsylvania, Philadelphia, PA; [20]University of Southern California, Los Angeles, CA; [21]University of Texas Southwestern Medical Center, Dallas, Texas; [22]University of Washington, Seattle, WA.
