# Supplementary material for: Mechanism of action biomarkers predicting response to AKT inhibition in the I-SPY 2 breast cancer trial
Source: NPJ Breast Cancer. 2020 Oct 2;6:48. doi: 10.1038/s41523-020-00189-2 (PMC7532145; doi:10.1038/s41523-020-00189-2)
Supplement: Supplementary file 2 — Reporting Summary Checklist FLAT [file 41523_2020_189_MOESM2_ESM.pdf]

nature research

Corresponding author(s): Denise Wolf

Last updated by author(s): Apr 23, 2020

Reporting Summary

Nature Research wishes to improve the reproducibility of the work that we publish. This form provides structure for consistency and transparency in reporting. For further information on Nature Research policies, see Authors & Referees and the Editorial Policy Checklist.

Statistics

For all statistical analyses, confirm that the following items are present in the figure legend, table legend, main text, or Methods section.

n/a	Confirmed	
☐	☒	The exact sample size (*n*) for each experimental group/condition, given as a discrete number and unit of measurement
☐	☒	A statement on whether measurements were taken from distinct samples or whether the same sample was measured repeatedly
☐	☒	The statistical test(s) used AND whether they are one- or two-sided<br>*Only common tests should be described solely by name; describe more complex techniques in the Methods section.*
☐	☒	A description of all covariates tested
☐	☒	A description of any assumptions or corrections, such as tests of normality and adjustment for multiple comparisons
☐	☒	A full description of the statistical parameters including central tendency (e.g. means) or other basic estimates (e.g. regression coefficient) AND variation (e.g. standard deviation) or associated estimates of uncertainty (e.g. confidence intervals)
☐	☒	For null hypothesis testing, the test statistic (e.g. *F*, *t*, *r*) with confidence intervals, effect sizes, degrees of freedom and *P* value noted<br>*Give P values as exact values whenever suitable.*
☐	☒	For Bayesian analysis, information on the choice of priors and Markov chain Monte Carlo settings
☒	☐	For hierarchical and complex designs, identification of the appropriate level for tests and full reporting of outcomes
☒	☐	Estimates of effect sizes (e.g. Cohen's *d*, Pearson's *r*), indicating how they were calculated

Our web collection on statistics for biologists contains articles on many of the points above.

Software and code

Policy information about availability of computer code

Data collection	Gene expression microarray intensity values are derived from Agilent Feature Extraction files. DataPrint, Agendia's proprietary software for extracting data from Agilent Feature Extraction files, performs log2 transformation on the gMeanSignal. The 75th quantile is then subtracted from the entire array, and a fixed value of 9.5 added.
Data analysis	Gene expression data and RPPA protein/phospho-protein analysis was performed using mostly standard statistical functions in the R computing environment (R Core Team (2013). R: A language and environment for statistical computing.) We also used the R package COMBAT (Johnston et. al, Biostatistics. 2007;8(1):118-27. doi: 10.1093/biostatistics/kxj037. PMID: 16632515) to remove 'batch' effects prior to combining microarrays from two different Agilent platforms (44K and 32K), and employed the R package rJAGS (Martyn Plummer (2019). rjags: Bayesian Graphical Models using MCMC. R package version 4-10. https://CRAN.R-project.org/package=rjags) to perform Bayesian regression. R scripts are available upon request.

For manuscripts utilizing custom algorithms or software that are central to the research but not yet described in published literature, software must be made available to editors/reviewers. We strongly encourage code deposition in a community repository (e.g. GitHub). See the Nature Research guidelines for submitting code & software for further information.

Data

Policy information about availability of data

All manuscripts must include a data availability statement. This statement should provide the following information, where applicable:

- Accession codes, unique identifiers, or web links for publicly available datasets
- A list of figures that have associated raw data
- A description of any restrictions on data availability

De-identified molecular and clinical data used in this study have been deposited in NCBI's Gene Expression Omnibus and are accessible through GEO SuperSeries accession number GSE150576 (https://www.ncbi.nlm.nih.gov/geo/query/acc.cgi?acc=GSE150576) and constituent Series accession numbers GSE149322 (https://www.ncbi.nlm.nih.gov/geo/query/acc.cgi?acc=GSE149322; gene expression data) and GSE150575 (https://www.ncbi.nlm.nih.gov/geo/query/acc.cgi?

acc=GSE150575; RPPA protein/phospho-protein data). Linear transformation parameters (gene expression) and normalization parameters (mean, sd per RPPA endpoint) to transform raw to normalized data are available as supplemental files on GEO as well, along with the normalized data.

Field-specific reporting

Please select the one below that is the best fit for your research. If you are not sure, read the appropriate sections before making your selection.

☒ Life sciences ☐ Behavioural & social sciences ☐ Ecological, evolutionary & environmental sciences

For a reference copy of the document with all sections, see nature.com/documents/nr-reporting-summary-flat.pdf

Life sciences study design

All studies must disclose on these points even when the disclosure is negative.

Sample size	This manuscript is a biomarker study of one arm of a phase 2 platform trial that has been published and referenced. Considerations around study design, including sample size, data exclusions, randomization and so forth are included in the efficacy publication: Chien AJ, Tripathy D, Albain KS, Symmans WF, Rugo HS, Melisko ME, Wallace AM, Schwab R, Helsten T, Forero-Torres A, Stringer-Reasor E, Ellis ED, Kaplan HG, Nanda R, Jaskowiak N, Murthy R, Godellas C, Boughey JC, Elias AD, Haley BB, Kemmer K, Isaacs C, Clark AS, Lang JE, Lu J, Korde L, Edmiston KK, Northfelt DW, Viscusi RK, Yee D, Perlmutter J, Hylton NM, Van't Veer LJ, DeMichele A, Wilson A, Peterson G, Buxton MB, Paoloni M, Clennell J, Berry S, Matthews JB, Steeg K, Singhrao R, Hirst GL, Sanil A, Yau C, Asare SM, Berry DA, Esserman LJ; I-SPY 2 Consortium. MK-2206 and Standard Neoadjuvant Chemotherapy Improves Response in Patients With Human Epidermal Growth Factor Receptor 2-Positive and/or Hormone Receptor-Negative Breast Cancers in the I-SPY 2 Trial. J Clin Oncol. 2020 Apr 1;38(10):1059-1069. doi: 10.1200/JCO.19.01027. Epub 2019 Feb 7. PubMed PMID:32031889; PubMed Central PMCID: PMC7106976.
Data exclusions	*Describe any data exclusions. If no data were excluded from the analyses, state so OR if data were excluded, describe the exclusions and the rationale behind them, indicating whether exclusion criteria were pre-established.*
Replication	*Describe the measures taken to verify the reproducibility of the experimental findings. If all attempts at replication were successful, confirm this OR if there are any findings that were not replicated or cannot be reproduced, note this and describe why.*
Randomization	*Describe how samples/organisms/participants were allocated into experimental groups. If allocation was not random, describe how covariates were controlled OR if this is not relevant to your study, explain why.*
Blinding	*Describe whether the investigators were blinded to group allocation during data collection and/or analysis. If blinding was not possible, describe why OR explain why blinding was not relevant to your study.*

Reporting for specific materials, systems and methods

We require information from authors about some types of materials, experimental systems and methods used in many studies. Here, indicate whether each material, system or method listed is relevant to your study. If you are not sure if a list item applies to your research, read the appropriate section before selecting a response.

Materials & experimental systems

n/a	Involved in the study	
☐	☒	Antibodies
☒	☐	Eukaryotic cell lines
☒	☐	Palaeontology
☒	☐	Animals and other organisms
☐	☒	Human research participants
☐	☒	Clinical data

Methods

n/a	Involved in the study	
☒	☐	ChIP-seq
☒	☐	Flow cytometry
☒	☐	MRI-based neuroimaging

Antibodies

Antibodies used	All antibodies used in the RPPA protein/phospho-protein arrays are described in the RPPA GEO data entry (in progress; link also within the gene expression series page for GSE149322 (https://www.ncbi.nlm.nih.gov/geo/query/acc.cgi?acc=GSE149322)).
Validation	*Describe the validation of each primary antibody for the species and application, noting any validation statements on the manufacturer's website, relevant citations, antibody profiles in online databases, or data provided in the manuscript.*

Human research participants

Policy information about studies involving human research participants

Population characteristics	See above

Recruitment

Describe how participants were recruited. Outline any potential self-selection bias or other biases that may be present and how these are likely to impact results.

Ethics oversight

Identify the organization(s) that approved the study protocol.

Note that full information on the approval of the study protocol must also be provided in the manuscript.

Clinical data

Policy information about clinical studies

All manuscripts should comply with the ICMJE guidelines for publication of clinical research and a completed CONSORT checklist must be included with all submissions.

Clinical trial registration

I-SPY 2 TRIAL (NCT01042379; IND 105139)

Study protocol

Protocols and such are described in I-SPY 2 efficacy papers including that for MK2206: J Clin Oncol. 2020 Apr 1;38(10):1059-1069. doi: 10.1200/JCO.19.01027. Epub 2019 Feb 7. PubMed PMID:32031889; PubMed Central PMCID: PMC7106976.

Data collection

See above

Outcomes

See above
